# Can robot-assisted gait training improve walking and activity abilities in persons with spinal cord injury? A systematic review and meta-analysis of randomized controlled trials

**DOI:** 10.3389/fneur.2026.1743421

**Published:** 2026-05-29

**Authors:** Xiaojuan Li, Jiliang Kang, Xiaohan Li, Weiping Liu, Min Tang, Xiaobo Chen

**Affiliations:** 1 Department of Neurological Rehabilitation, Ningbo Rehabilitation Hospital, Ningbo, China; 2 Ningbo Key Laboratory of Brain Cognitive Rehabilitation, Ningbo Rehabilitation Hospital, Ningbo, China; 3 School of Rehabilitation, Gannan Medical University, Ganzhou, China; 4 The Second Affiliated Hospital of Gannan Medical University, Ganzhou, China

**Keywords:** gait training, meta-analysis, randomized controlled trials, robot-assisted gait training, spinal cord injury

## Abstract

**Background:**

Spinal cord injury (SCI) can lead to substantial impairments in walking and mobility, and traditional rehabilitation methods face limitations such as high labor intensity and insufficient training intensity and precision. While robot-assisted gait training is widely used, its therapeutic advantages over conventional rehabilitation remain controversial.

**Objective:**

Through systematic reviews and meta-analysis, this study quantitatively synthesizes existing randomized controlled trial (RCT) evidence to determine the efficacy of robot-assisted gait training on walking and mobility abilities in persons with SCI.

**Methods:**

A comprehensive search was conducted across PubMed, Embase, Cochrane Library, Web of Science, and China National Knowledge Infrastructure (CNKI) databases from their inception to June 19, 2025. We included RCTs comparing robot-assisted gait training with conventional rehabilitation. Two researchers independently performed literature screening, data extraction, and risk of bias assessment. Meta-analysis was conducted using RevMan 5.4 software to calculate standardized mean differences (SMDs) and their 95% confidence intervals (CIs).

**Results:**

A total of 8 RCTs involving 241 participants were included. Meta-analysis results showed: (1) Regarding walking ability, the robot-assisted gait training group demonstrated significant improvements in the 6-min walk test distance (SMD = 0.57, 95% CI: 0.12–1.03, *p* = 0.01) and Spinal Cord Injury Walking Index II score (SMD = 0.49, 95% CI: 0.13–0.84, *p* = 0.007); (2) Regarding functional independence, the robot-assisted gait training group demonstrated superior improvement in functional independence scores (SMD = 0.39, 95% CI: 0.05–0.72, *p* = 0.02); (3) Regarding lower limb muscle strength, no significant difference was observed between the two groups in the improvement of lower limb motor scores (SMD = 0.03, 95% CI: −0.27–0.34, *p* = 0.84).

**Conclusion:**

Robot-assisted gait training effectively improves functional walking endurance, gait independence, and overall function in persons with SCI. Its core mechanism lies in promoting task-specific motor learning and neuromuscular control, rather than enhancing muscle strength. It is recommended that this technology be adopted as a primary functional improvement strategy in clinical practice, combined with targeted strength training to optimize rehabilitation outcomes.

## 1 Introduction

Spinal cord injury (SCI) is a life-altering neurological condition arising from damage to the spinal cord’s neural pathways. The initial injury triggers a complex pathophysiological cascade, including a destructive secondary phase of inflammation and ischemia that expands the neural damage (1). This process disrupts ascending sensory and descending motor tracts, culminating in a wide spectrum of impairments affecting motor, sensory, and autonomic systems (2). The functional outcome is largely dictated by the injury’s level and severity, often classified by the ASIA Impairment Scale (3). Injuries to the thoracic, lumbar, or sacral regions specifically compromise lower limb function, leading to paraparesis or paraplegia. With a global prevalence exceeding 15 million (4), SCI represents a substantial global health burden, imposing immense challenges on individuals, families, and healthcare systems.

For those with lower-level SCI, the loss of ambulatory function is one of the most debilitating consequences (5). When independent walking is not possible, many individuals rely on wheelchairs for mobility. Although wheelchair use can enhance environmental mobility and independence, loss of ambulatory function is still associated with secondary health complications, reduced community participation, and increased rehabilitation needs. Chronic immobility contributes to musculoskeletal degradation, including muscle atrophy and osteoporosis; cardiovascular deconditioning; and an increased risk of metabolic syndrome and pressure ulcers. The psychosocial impact is equally profound, often leading to social isolation, vocational barriers, and diminished community participation (6, 7). This complex interplay of physical and psychological challenges significantly reduces overall quality of life and can hinder motivation for rehabilitation (8, 9). Consequently, restoring walking ability is a paramount therapeutic priority, aimed not just at improving mobility but also at mitigating these secondary complications and fostering greater independence.

The foundational principle of gait rehabilitation in SCI is activity-dependent neuroplasticity—the central nervous system’s capacity for reorganization in response to intensive, task-specific training (10). For decades, conventional rehabilitation paradigms, such as Body-Weight Supported Treadmill Training (BWSTT) and therapist-facilitated overground training, have been the clinical standard. These methods leverage neuroplastic principles by enabling early mobilization and the repetitive practice of stepping (11, 12). While their efficacy is supported by evidence, these conventional approaches are beset by significant practical limitations. They are exceptionally labor-intensive, often requiring multiple therapists to manually assist a single person. This incurs substantial costs and places considerable physical strain on clinicians (13). Furthermore, manual assistance is inherently variable, making it difficult to achieve the high dosage and consistent, biomechanically precise movements considered optimal for driving neuroplasticity.

In response to these limitations, robot-assisted gait training (RAGT) has emerged as a promising technological intervention. These systems include both wearable robotic exoskeletons for overground walking and treadmill-based robotic gait orthoses, such as Lokomat, that provide guided lower-limb movement during gait training (14). Their primary advantage lies in the ability to deliver a high volume of repetitive stepping practice with a consistent and precise gait pattern, far exceeding manual capabilities. By providing reliable body support and actively guiding the joints, exoskeletons reduce the physical burden on therapists, allowing them to focus on other critical aspects of therapy (15). Moreover, integrated sensors provide objective, real-time data on gait parameters (e.g., speed, step length, kinematics), enabling data-driven progress monitoring (16). The proposed therapeutic mechanisms for RAGT include enhanced neuroplasticity via consistent afferent feedback, prevention of learned non-use, and improved motor control.

Despite the compelling theoretical advantages and rapid clinical adoption of robotic exoskeletons, the evidence for their superiority over conventional therapies remains equivocal. A growing body of randomized controlled trials (RCTs) has investigated the effects of RAGT, but their findings have been inconsistent. Some studies demonstrate significant benefits in gait speed, endurance, and balance, while others report no clear advantage when compared to intensive conventional training (17–20). This heterogeneity in outcomes is likely influenced by substantial variations across studies in participant characteristics (e.g., time since injury, lesion severity), intervention protocols (e.g., training duration, frequency), and the type of robotic device used. These conflicting results, often from trials with small sample sizes, make it challenging for clinicians to formulate clear, evidence-based guidelines for the use of this technology.

Although several recent reviews have examined exoskeleton-based rehabilitation after spinal cord injury, important gaps remain (21–24). Some reviews have focused specifically on dose and dosage parameters for overground exoskeleton training (24), whereas others have pooled broader evidence bases that included both randomized and non-randomized controlled studies (21). In addition, some recent reviews have emphasized outcome domains such as walking balance, respiratory function, lower extremity function, ambulation, or quality of life, rather than the specific combination of clinically interpretable walking and functional outcomes examined in the present study (22, 23). Therefore, the present systematic review and meta-analysis was designed to provide a focused synthesis of randomized controlled trials comparing robot-assisted gait training with conventional rehabilitation, with particular attention to the 6-min walk test, Walking Index for Spinal Cord Injury II, Lower Extremity Motor Score, and Functional Independence Measure. By pooling data from these trials, we aimed to clarify the comparative effects of this intervention and to provide a more focused evidence base for clinical decision-making in SCI rehabilitation.

## 2 Methods

### 2.1 Protocol and registration

This systematic review and meta-analysis was conducted and reported in accordance with the Preferred Reporting Items for Systematic Reviews and Meta-analysis (PRISMA) guidelines (25). The study protocol was registered in the International Prospective Register of Systematic Reviews (PROSPERO) under the registration number CRD420251135682. Because the literature search was completed before formal registration, the PROSPERO registration should be considered retrospective.

### 2.2 Literature search

A systematic literature search was conducted in PubMed, Embase, the Cochrane Library, Web of Science, and China National Knowledge Infrastructure (CNKI) from database inception to June 19, 2025, without language restrictions. The search strategy combined controlled vocabulary terms (e.g., MeSH terms in PubMed and equivalent subject headings in other databases) with free-text keywords related to spinal cord injury, robot-assisted gait training, and gait or walking outcomes. Synonyms within each concept were combined using the Boolean operator OR, and different concept groups were combined using AND. The search syntax was adapted to the requirements of each database. The complete database-specific search strategies, including subject headings, free-text terms, Boolean operators, and any applied limits, are provided in the Supplementary Appendix.

### 2.3 Study selection

All retrieved citations were imported into EndNote X9 for management and removal of duplicate records. Subsequently, two reviewers (XL and JK) independently screened the titles and abstracts of the remaining articles against the predefined eligibility criteria. The full texts of potentially relevant studies were then retrieved and assessed for final inclusion. Any disagreements between the two reviewers during the screening process were resolved through discussion or, if necessary, by consulting a third reviewer (XC).

Studies were included if they met the following criteria:

(1) Population: The study participants were adults diagnosed with spinal cord injury.
(2) Intervention: The intervention consisted of robot-assisted gait training delivered using either a wearable robotic exoskeleton or a treadmill-based robotic gait orthosis.
(3) Comparison: The control group received conventional rehabilitation training.
(4) Outcomes: The study reported at least one of the following outcome measures: Lower Extremity Motor Score (LEMS), 6-Minute Walk Test (6MWT), Walking Index for Spinal Cord Injury II (WISCI II), or Functional Independence Measure (FIM).
(5) Study Design: The study was a randomized controlled trial (RCT).

Exclusion criteria were as follows: (1) non-RCT designs; (2) interventions using robotic devices outside the predefined scope of exoskeleton- or orthosis-based gait training, such as end-effector systems; (3) control groups that received no intervention or were on a waitlist; and (4) studies for which the full text or relevant data were unavailable.

### 2.4 Data extraction

Two reviewers independently extracted data from the included studies using a standardized data extraction form. The extracted information was then cross-checked for accuracy and consistency. Any discrepancies were resolved by discussion or through arbitration by a third reviewer.

The following data were extracted from each study:

(1) First author and year of publication
(2) Country of origin
(3) Participant characteristics (e.g., age, time since injury)
(4) Sample size for intervention and control groups
(5) Detailed descriptions of the intervention and control conditions
(6) Outcome measures, including baseline and post-intervention mean and standard deviation (SD) values for each outcome

### 2.5 Quality assessment

The methodological quality and risk of bias of the included RCTs were independently assessed by two reviewers using the Cochrane Risk of Bias tool (RoB 1). The assessment was facilitated using Review Manager (RevMan, version 5.4) software. Each study was evaluated across seven domains: (1) random sequence generation, (2) allocation concealment, (3) blinding of participants and personnel, (4) blinding of outcome assessment, (5) incomplete outcome data, (6) selective reporting, and (7) other bias. Each domain was judged as having a “low risk,” “high risk,” or “unclear risk” of bias. Disagreements in assessment were resolved through discussion or consultation with a third reviewer until a consensus was reached.

In addition, the certainty of evidence for the main outcomes was assessed using the Grading of Recommendations Assessment, Development and Evaluation (GRADE) approach. The certainty of evidence was rated as high, moderate, low, or very low according to the domains of risk of bias, inconsistency, indirectness, imprecision, and publication bias. The detailed GRADE evidence profile is presented in Supplementary Table S2.

### 2.6 Statistical analysis

This study employed the Review Manager 5.4 software recommended by the Cochrane Collaboration for the meta-analysis. Regarding continuous outcome indicators (FIM scores, WISCI II scores, etc.), the standardized mean difference (SMD) was selected as the effect size, and the 95% confidence interval (CI) was calculated; risk ratios (RRs) and corresponding 95% CIs were employed for analyzing dichotomous outcome (adverse events). The inter-group heterogeneity was tested using Cochran’s Q statistics and *I*^2^. If there is no heterogeneity among the groups (Q test shows *p* > 0.05 or *I*^2^ < 50%), then the fixed effect model will be used. Conversely, in the case where the Q test results are significant (*p* < 0.05 or *I*^2^ > 50%), then the random effects model is used (26). The indicator of significant heterogeneity is that *I*^2^ exceeds 50%. A *p* value < 0.05 is regarded as statistically significant.

## 3 Results

### 3.1 Literature search process results

**Figure 1:**
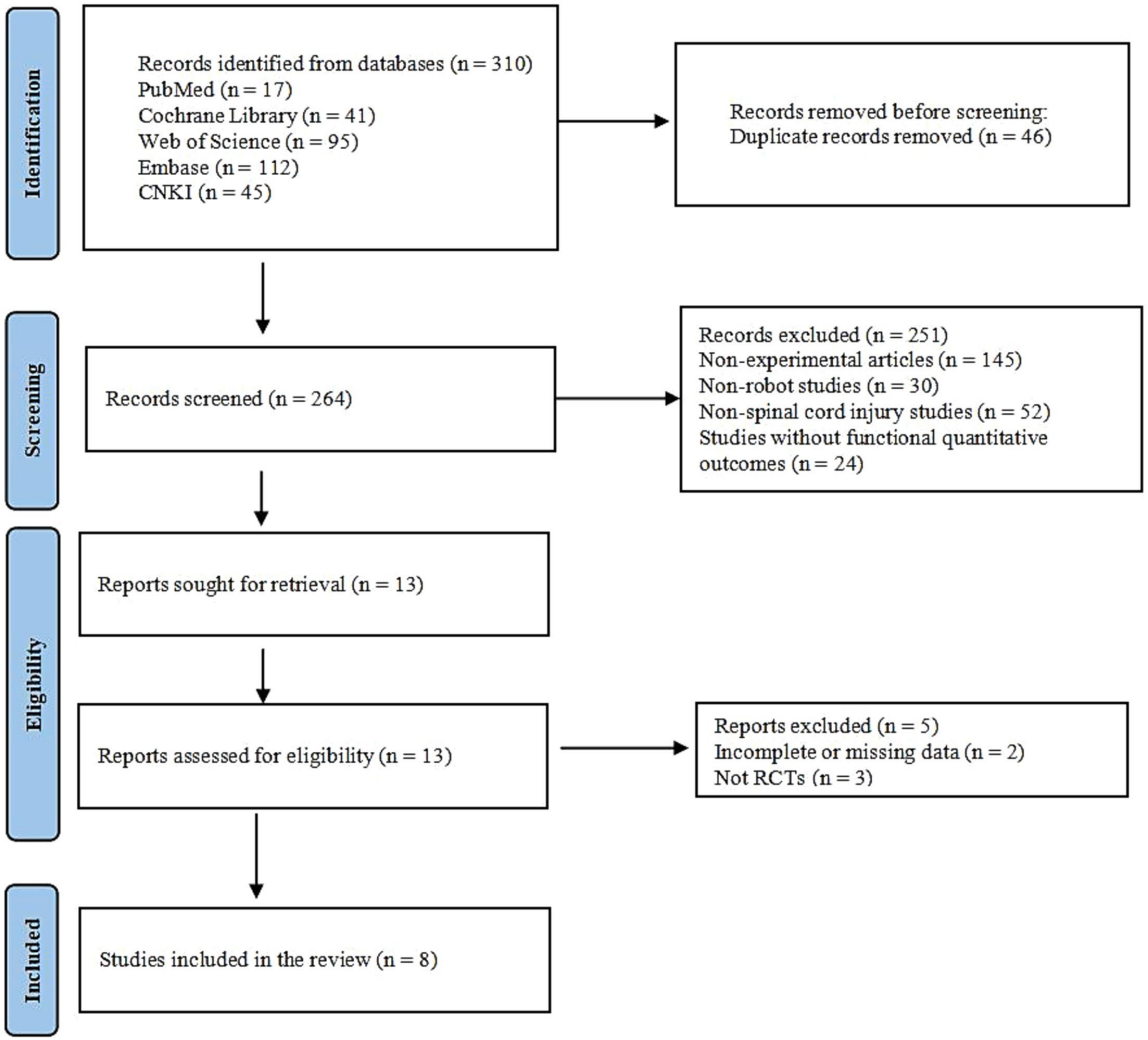
PRISMA flow diagram of study selection. PRISMA, Preferred Reporting Items for Systematic Reviews and Meta-Analysis.

The study selection process is summarized in the PRISMA flow diagram (Figure 1). A total of 310 records were identified through database searching. After the removal of 46 duplicate records, 264 records were screened. Following title and abstract screening, 13 full-text reports were retrieved and assessed for eligibility. Of these, five reports were excluded, and eight randomized controlled trials were ultimately included in the systematic review and meta-analysis.

### 3.2 Study characteristics

The key characteristics of the eight included studies are summarized in Table 1. These RCTs were published between 2014 and 2023 and originated from four different countries. Seven studies were published in English and one in Chinese. In all included trials, the intervention group received robot-assisted gait training delivered either through wearable exoskeletons or treadmill-based robotic gait orthoses. The specific protocols varied, with two studies (27, 28) utilizing Exoskeleton-Assisted Walking (EAW), one (29) employing Robotic Locomotor Training plus Overground Therapy (LKOGT), three (30–32) using Robot-Assisted Gait Training (RAGT), and two (33, 34) describing their intervention as general robotic gait training. The control groups uniformly underwent conventional rehabilitation, which typically included lower limb strength training and other standard physical therapy exercises. The treatment frequency across the studies ranged from three to five sessions per week. Regarding the outcome measures, six studies (27–30, 33, 34) reported the Lower Extremity Motor Score (LEMS), four (29–31, 34) reported the Walking Index for Spinal Cord Injury II (WISCI II), three (28, 29, 34) reported the 6-Minute Walk Test (6MWT), and three (29, 31, 32) reported the Functional Independence Measure (FIM).

**Table 1:** Characteristics of the included studies.

Author (year)	Country	Test types	Age (years) (M ± SD)	Experimental group (*N*)	Control group (*N*)	Intervention protocol	Control scheme	Intervention protocol intensity	Outcome
Xiang et al. (2023) (27)	China	RCT	E:36.6 ± 11.7 C:37.7 ± 12.6	20	20	EAW	Conventional training	16 times in 4 weeks. 16 times (1 time /day)	LEMS
Xiang et al. (2021) (28)	China	RCT	E:39.8 ± 12.2 C:36.6 ± 11.8	9	9	EAW	Conventional training	16 times in 4 weeks. 16 times (4 times/week)	LEMS, 6MWT
Wu et al. (2018) (33)	USA	RCT	E:48.4 ± 13.5 C:48.1 ± 4.9	8	8	Robotic treadmill training	Conventional training	18 times in 6 weeks (3 times/week)	LEMS
Mıdık et al. (2020) (30)	Turkey	RCT	E:35.4 ± 12.1 C:37.9 ± 10.0	15	15	RAGT	Conventional training	15 times in 5 weeks (3 times/week)	LEMS, WISCI II
Gil-Agudo et al. (2023) (34)	Spain	RCT	E:41 ± 12.39 C:51.8 ± 11.93	11	10	Robotic gait training	Conventional training	15 times in 5 weeks (3 times/week)	LEMS, 6MWT, WISCI II
Çinar et al. (2021) (31)	Turkey	RCT	E:32.9 ± 11.4 C:36.9 ± 12.6	17	20	RAGT	Conventional training	40 times in 8 weeks (1 time/day, 5 times/week)	WISCI II, FIM
Esclarín-Ruz et al. (2014) (29)	Spain	RCT	E:43.6 ± 12 C:44.9 ± 7.0	21	21	LKOGT	Conventional training	40 times in 8 weeks	LEMS, 6MWT, WISCI II, FIM
Yang et al. (2022) (32)	China	RCT	E:36.03 ± 10.15 C:34.86 ± 9.74	31	31	RAGT	Conventional training	40 times in 8 weeks (5 times/week)	FIM

E, Experimental group; C, Control group; EAW, Exoskeleton-assisted walking; RAGT, Robot-assisted gait training; LKOGT, Robotic locomotor training plus overground therapy; LEMS, Lower extremity motor score; 6MWT, 6-min walk test (distance); WISCI II, Walking Index for Spinal Cord Injury II; FIM, Functional Independence Measure.

### 3.3 Quality assessment

**Figure 2:**
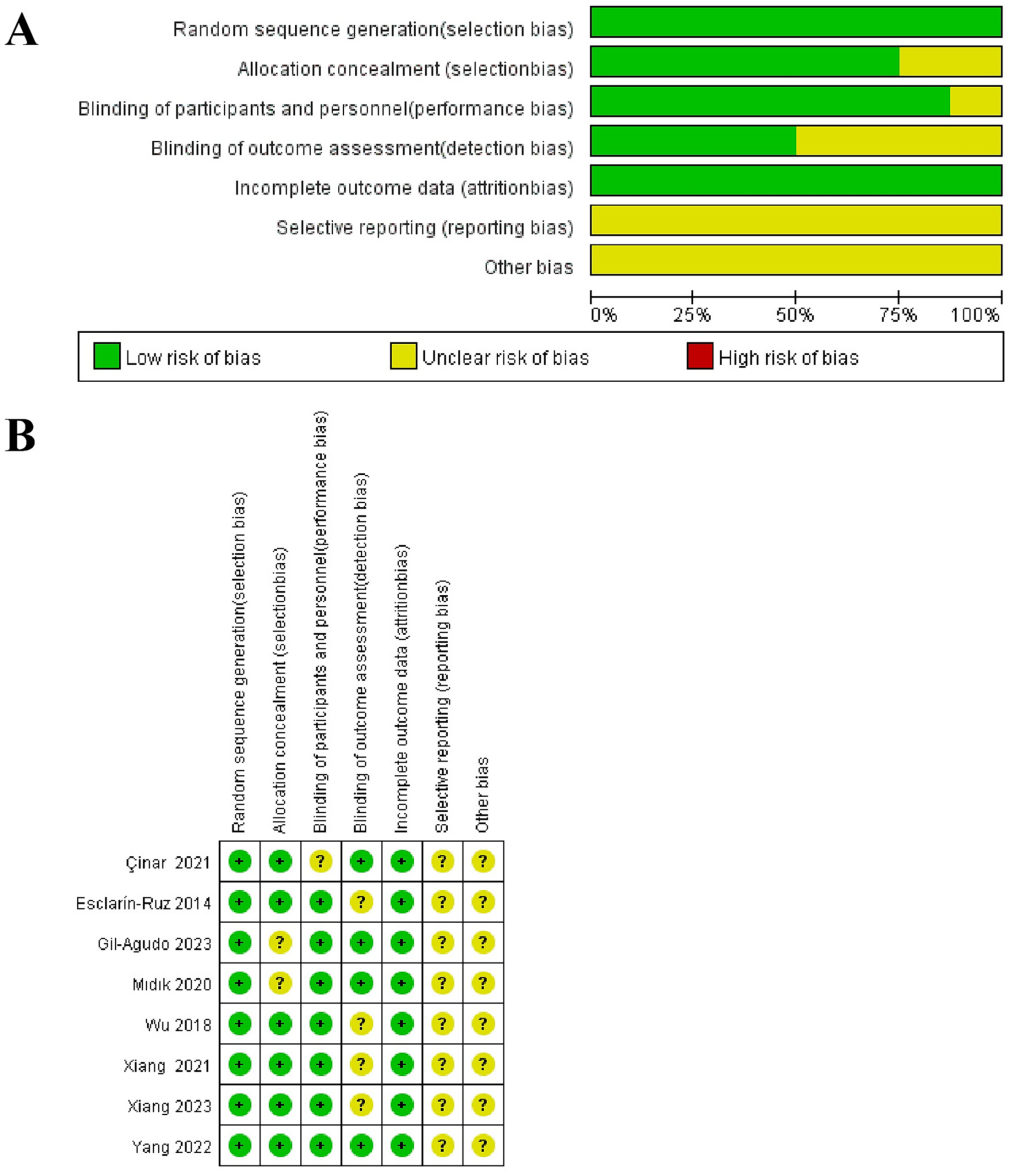
Risk of bias of the included randomized controlled trials. **(A)** Risk of bias graph. **(B)** Risk of bias summary.

The results of the risk of bias assessment for the eight included RCTs are presented in Figure 2. All included studies (100%) were judged to be at low risk of bias for random sequence generation. For allocation concealment, six studies (75%) (27–29, 31–33) provided a clear description and were rated at low risk, while the remaining two (25%) (30, 34) provided insufficient information, resulting in an unclear risk. Regarding blinding of participants and personnel, seven studies (87.5%) (27–30, 32–34) were deemed to be at low risk. In the assessment of blinding of outcome assessors, four studies (50%) (30–34) were rated at low risk. All eight studies (100%) were judged to have a low risk of bias from incomplete outcome data (attrition bias). Finally, the risk of bias from selective reporting was unclear across all eight studies (100%), as their protocols were not available for comparison.

In addition, the certainty of evidence for the main outcomes was evaluated using the GRADE approach. The detailed evidence profile is presented in Supplementary Table S2. Overall, the certainty of evidence ranged from low to moderate, with downgrading mainly due to imprecision, moderate heterogeneity in the 6MWT outcome, and risk-of-bias concerns for assessor-dependent functional outcomes.

### 3.4 Meta-analysis results

#### 3.4.1 Analysis of 6-min walk test

**Figure 3:**
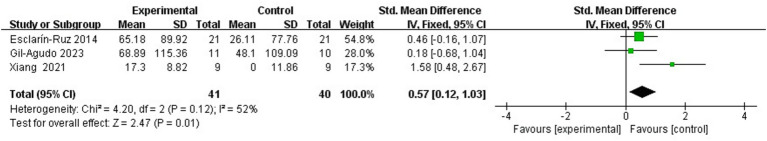
Forest plot of robot-assisted gait training versus conventional rehabilitation for the 6-Minute Walk Test (6MWT). SD, standard deviation; 95% CI, 95% confidence interval; Std, standard.

Three studies (28, 29, 34), encompassing 81 participants, provided data on the 6MWT. The forest plot is presented in Figure 3. The pooled analysis showed that robot-assisted gait training led to a statistically significant increase in walking distance compared with the control group, with moderate heterogeneity observed among the studies (SMD = 0.57; 95% CI = 0.12 to 1.03; *p* = 0.01; *I*^2^ = 52%). The observed SMD = 0.57 in 6MWT corresponds to an actual walking distance increase of approximately 45–50 meters, exceeding the reported MCID threshold of 30 meters, indicating clinical significance. Due to moderate heterogeneity, a subgroup analysis was performed based on the type of robot-assisted gait system.

##### 3.4.1.1 Wearable EAW (exoskeleton-assisted walking)

This subgroup included the studies by Xiang et al. (28) and Gil-Agudo et al. (34). Both studies showed improved walking distance in the robotic exoskeleton group compared with the control group, although the magnitude of improvement varied across studies.

##### 3.4.1.2 Treadmill-based RAGT (robot-assisted gait training)

This subgroup included the study by Esclarín-Ruz et al. (29). Participants in the robotic training group exhibited a greater increase in walking distance than those in the control group (A1: 187.5 ± 103.8 m, B1: 157.5 ± 89.5 m), suggesting that treadmill-based RAGT may facilitate greater short-term gains in walking endurance through high-repetition gait training.

#### 3.4.2 Analysis of walking index for spinal cord injury II

**Figure 4:**
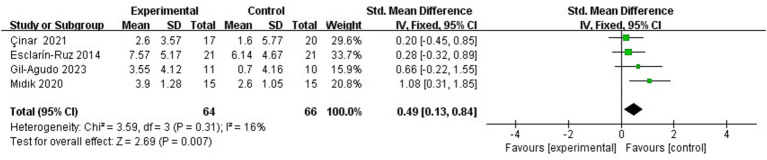
Forest plot of robot-assisted gait training versus conventional rehabilitation for the Walking Index for Spinal Cord Injury II (WISCI II). SD, standard deviation; 95% CI, 95% confidence interval; Std, standard.

Four studies (29–31, 34) with a total of 130 participants reported on the WISCI II. As shown in Figure 4, the meta-analysis revealed that the robot-assisted gait training group achieved a statistically significant improvement in WISCI II scores compared with the control group, with low heterogeneity (SMD = 0.49; 95% CI = 0.13 to 0.84; *p* = 0.007; *I*^2^ = 16%). The improvement of SMD = 0.49 in WISCI-II corresponds to an increase of approximately one level in daily walking independence, reaching a level that represents a meaningful improvement in patients’ functional self-care.

#### 3.4.3 Analysis of lower extremity motor score

**Figure 5:**
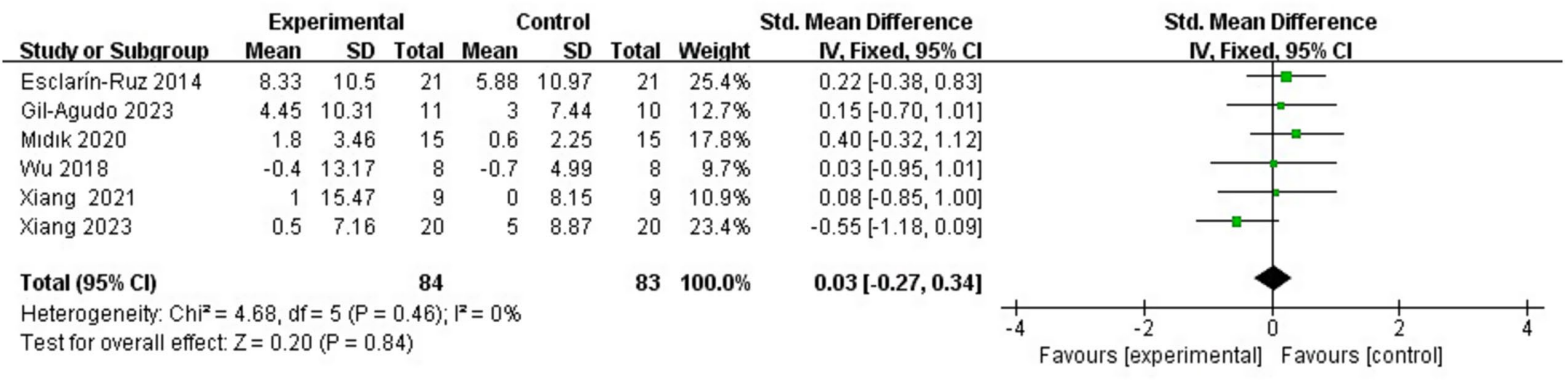
Forest plot of robot-assisted gait training versus conventional rehabilitation for the lower extremity motor score (LEMS). SD, standard deviation; 95% CI, 95% confidence interval; Std, standard.

Six studies (27–30, 33, 34), including a total of 167 participants, reported LEMS outcomes. As shown in the forest plot in Figure 5, the pooled analysis indicated that there was no statistically significant difference in LEMS improvement between the robot-assisted gait training group and the control group. No heterogeneity was detected for this outcome (SMD = 0.03; 95% CI = −0.27 to 0.34; *p* = 0.84; *I*^2^ = 0%).

#### 3.4.4 Analysis of functional Independence measure

**Figure 6:**
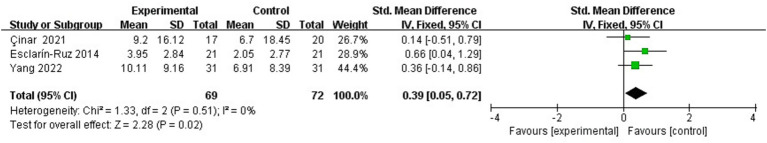
Forest plot of robot-assisted gait training versus conventional rehabilitation for the functional independence measure (FIM). SD, standard deviation; 95% CI, 95% confidence interval; Std, standard.

Three studies (29, 31, 32) with a combined total of 141 participants provided data on FIM scores. As shown in Figure 6, the analysis demonstrated that participants in the robot-assisted gait training group had statistically significantly greater improvements in FIM scores than those in the control group. No heterogeneity was observed across the studies for this outcome (SMD = 0.39; 95% CI = 0.05 to 0.72; *p* = 0.02; *I*^2^ = 0%).

#### 3.4.5 Analysis of different types of spinal cord injury

Two studies (31, 32) investigated persons with complete SCI (cSCI), whereas the remaining six studies focused primarily on persons with incomplete SCI (iSCI). Stratified analyses of FIM and WISCI II showed that, among persons with cSCI, no significant difference was observed between the RAGT group and the control group (*p* > 0.05), whereas among persons with iSCI, the experimental group showed significantly greater improvements than the control group (*p* < 0.05).

## 4 Discussion

### 4.1 Summary of principal findings and central thesis

This systematic review and meta-analysis was conducted to resolve the uncertainty surrounding the efficacy of robot-assisted gait training for persons with SCI by quantitatively synthesizing the evidence from all available randomized controlled trials. The principal finding of our study is a critical and insightful thesis: the therapeutic effects of robot-assisted gait training exhibit a clear functional-strength dissociation. Specifically, our analysis demonstrates that this advanced intervention is significantly superior to conventional rehabilitation for improving functional walking capacity, as measured by the 6MWT and the WISCI II, and for enhancing overall functional independence, as reflected by the FIM. In stark contrast, an equally robust finding was that this intervention conferred no additional benefit over conventional therapy in improving isolated lower extremity muscle strength, as measured by the LEMS. This collection of findings provides a more precise and profound perspective: the primary therapeutic value of robot-assisted gait systems does not appear to stem from the physiological restoration of muscle power, but is instead deeply rooted in their unique capacity to facilitate task-specific motor learning, promote neuromuscular re-education, and remodel locomotor patterns. The subsequent discussion will provide an in-depth dissection of the potential neurophysiological, biomechanical, and motor learning mechanisms that likely underlie each of these distinct outcomes.

### 4.2 Mechanisms of enhanced walking endurance: in-depth interpretation of the 6MWT results

A key positive finding from this meta-analysis was the statistically significant increase in 6MWT distance (SMD = 0.57) following robot-assisted gait training. The 6MWT is the gold standard for assessing submaximal aerobic endurance, and its improvement suggests complex underlying mechanisms (35). The most central of these is likely the enhancement of neuromuscular efficiency and the corresponding reduction in the metabolic cost of walking. Gait patterns following SCI are often characterized by compensatory movements, such as hip hiking or circumduction, which are biomechanically inefficient and energetically demanding (10, 36). Robot-assisted gait systems, through their precise trajectory control, compel the user to practice a kinematically optimized, physiological gait pattern for thousands of repetitions. This intensive, patterned practice helps the central nervous system (CNS) to suppress inefficient compensatory strategies and adopt a more economical motor program (37, 38). As the movement pattern becomes more efficient, the metabolic cost per step is reduced, allowing the patient to walk for a longer duration or greater distance before the onset of fatigue.

A second, complementary mechanism is the improvement in cardiopulmonary conditioning. For many individuals with severe SCI, achieving and sustaining a target heart rate for effective aerobic training during conventional overground therapy can be challenging due to balance limitations and the need for constant therapist intervention (39). RAGT, in particular, provides a stable and continuous environment that facilitates sustained, rhythmic lower limb movement. This provides a consistent cardiovascular stimulus that may be difficult to otherwise achieve (40), leading to improvements in cardiovascular fitness and aerobic capacity, which are direct physiological determinants of performance on an endurance test such as the 6MWT.

It is crucial, however, to acknowledge the moderate heterogeneity observed in the 6MWT outcome (*I*^2^ = 52%). This indicates that the magnitude of the effect on endurance varied across the included studies. The most plausible explanation for this variability lies in the diversity of intervention protocols and baseline patient characteristics. For instance, the type of robotic system used—such as a stationary, treadmill-based RAGT versus a mobile, overground EAW device—may provide different levels of aerobic stimulus (41). Specifically, treadmill-based RAGT provides high-repetition, stable, and guided gait patterns that facilitate substantial short-term improvements in walking endurance, particularly for patients with lower baseline functional levels. In contrast, wearable EAW devices allow overground walking and balance challenges, enhancing task-specific training but showing relatively smaller short-term gains in walking distance, potentially due to differences in training intensity, frequency, and patient baseline characteristics. Likewise, variations in training frequency (3 vs. 5 sessions per week) and total intervention duration would inevitably impact adaptations related to endurance (30). Furthermore, the baseline functional level of the participants is a significant confounding factor; a patient with very poor initial endurance has substantially more room for improvement than one who is already a limited community ambulator. The interplay of these factors likely accounts for the observed heterogeneity.

### 4.3 Mechanisms of improved walking Independence: in-depth interpretation of the WISCI II results

Another key positive finding of this review was the significant improvement in the WISCI II scores (SMD = 0.49), with low inter-study heterogeneity (*I*^2^ = 16%). The WISCI II is a hierarchical scale (42) that quantifies the level of physical assistance and assistive devices required for walking. An improvement in this score is a direct indicator of enhanced walking independence. This highly consistent result is deeply rooted in the principles of motor skill acquisition and consolidation. Independent walking should be conceptualized as a complex motor skill. Within this framework, the robot-assisted gait system functions as a dynamic scaffold. In the initial stages of training, it provides maximal external stability and guidance, creating a safe, low-error environment where the patient can focus on practicing the temporal and spatial sequences of gait (43, 44). This high volume of successful practice is a cornerstone of motor learning, leading to the consolidation of a more robust and independent motor program for locomotion (45, 46). As training progresses, many advanced robotic systems can operate in an “assist-as-needed” mode, systematically reducing the level of support and encouraging more active participation from the patient. This facilitates the transfer and generalization of the learned skill, ultimately reducing the reliance on walkers, crutches, or therapist assistance. The very low heterogeneity strongly suggests that promoting walking independence is a highly reliable and predictable therapeutic effect of this training paradigm. However, it must be acknowledged that the lack of blinding for outcome assessors in 50% of the included studies may introduce detection bias, particularly for clinician-rated scales like WISCI II. This suggests that the positive results in functional independence should be interpreted with caution.

Furthermore, proprioceptive re-calibration and the enhancement of sensorimotor integration represent another critical underlying mechanism. An SCI often disrupts the flow of afferent information, including proprioception (47). The precise, repetitive, and consistent movements enforced by the system provide the CNS with a powerful, coherent, and prolonged stream of proprioceptive input from the lower limb joints and muscles (48, 49). This sustained and structured sensory information may help to re-calibrate the CNS’s internal model (or body schema) of the lower limbs, improving the integration of sensory feedback with motor commands. Enhanced sensorimotor integration is fundamental for improving dynamic balance control, which is a key factor in reducing the need for external support during ambulation, the very construct measured by the WISCI II scale.

### 4.4 Mechanisms of global functional gain: in-depth interpretation of the FIM results

Our analysis also revealed a significant improvement in the overall measure of functional independence, the FIM score (SMD = 0.39), with zero heterogeneity (I^2^ = 0%). The FIM is a comprehensive instrument that assesses a patient’s self-care ability in both motor and cognitive domains (50). The improvement in FIM scores following robotic training can be attributed to both direct and indirect mechanisms. The direct mechanism is the transfer of motor skills. The motor subscale of the FIM includes tasks such as transfers, locomotion, and stair climbing. The gains in dynamic trunk control, standing balance, and lower limb coordination achieved during intensive robotic gait training have a direct and positive transfer to these other essential daily functional tasks (51, 52). For example, a patient who improves their standing balance during gait training will undoubtedly be safer and more independent when performing a standing pivot transfer from a bed to a chair.

The indirect mechanism operates through a positive psychosocial effect, namely an increase in self-efficacy. For an individual who has been reliant on a wheelchair, the experience of standing and walking again, even within a robotic device, can be profoundly empowering (53). This experience can dramatically increase a patient’s self-efficacy and reduce the fear of falling, which is a major psychological barrier to functional independence (54). This newfound confidence often breaks the cycle of “learned non-use” and motivates patients to attempt more activities of daily living on their own, creating a positive feedback loop of practice, success, and further attempts. This global effect on motivation and participation is then captured by an overall improvement in the FIM score. The finding of zero heterogeneity suggests that this is a remarkably consistent and robust secondary benefit of regaining ambulatory function through robotic training.

### 4.5 The critical distinction: in-depth interpretation of the null LEMS results

In striking contrast to the positive functional outcomes, our meta-analysis found unequivocally that robot-assisted gait training was not superior to conventional therapy for improving LEMS (SMD = 0.03), a finding reinforced by zero heterogeneity (I^2^ = 0%). This null finding is not an indication of the failure of the intervention, but rather a crucial insight into its mechanical and physiological specificity. The most fundamental explanation lies in the Principle of Specificity in exercise physiology. LEMS is a measure of maximal voluntary muscle contraction in isolated muscle groups (55). The physiological stimulus required to augment this type of pure strength is progressive overload, wherein muscles are challenged to contract against a gradually increasing resistance (56–58). Robot-assisted gait systems are primarily designed for neuromuscular re-education through high-repetition, low-resistance practice (59). The training is specific to the skill and endurance of walking, not the maximal strength of the leg muscles. This dissociation between functional gait improvement and muscle strength gain can be further elucidated by the intrinsic neuroplasticity of the spinal cord. Robot-assisted gait training provides repetitive, rhythmic sensory feedback that effectively activates the Spinal Central Pattern Generators (CPGs), thereby enhancing gait coordination and rhythmic consistency. However, because the robotic actuators provide significant motor assistance to ensure trajectory accuracy, the patient’s voluntary motor unit recruitment—and the subsequent muscle fiber hypertrophy (specifically Type II fibers) required for strength—may be insufficient to manifest as an increase in LEMS (60). This reinforces the “task-specific” nature of such rehabilitation: the intervention optimizes the global movement pattern and neural timing without necessarily demanding or inducing raw muscular force. To use an analogy, a marathon runner’s training (specificity: endurance) does not make them a proficient powerlifter (specificity: maximal strength). Since conventional therapy programs often include specific progressive resistance exercises (PRE), it is logical that the control group would see comparable gains in this specific domain.

On a deeper neurophysiological level, this finding can be understood through the lens of restitution versus compensation in neural recovery. Recovery after SCI can be broadly categorized into two pathways. Restitution refers to the actual physiological repair or restoration of damaged neural pathways (e.g., remyelination, axonal sprouting) (61, 62), which could plausibly lead to an increase in voluntary muscle power as measured by LEMS. Compensation, on the other hand, refers to the CNS learning to use remaining, intact neural pathways more efficiently to accomplish a task (63). Robot-assisted gait training, with its emphasis on massed practice of a specific motor pattern, is an exceptionally powerful tool for driving compensatory neural strategies (64). It effectively teaches the brain and spinal cord how to generate a functional output (walking) by optimizing the use of available neural resources. The null result for LEMS, especially with zero heterogeneity, strongly suggests that the primary benefits of RAGT are mediated through these compensatory mechanisms, rather than through a fundamental restitution of corticospinal tract integrity that would be required to improve volitional muscle force generation. Therefore, the lack of a superior effect on LEMS is an expected outcome based on the specific nature of the intervention.

### 4.6 Synthesis and clinical implications

In summary, this meta-analysis indicates that RAGT is a specific tool for functional motor learning rather than general strengthening. Our stratified analysis underscores this: while persons with iSCI showed significantly greater improvements in FIM and WISCI-II due to the activation of residual neural pathways, those with cSCI showed no significant gains over controls.

Consequently, clinical decisions must be goal-oriented and injury-specific. For cSCI, RAGT may require adjunct therapies like functional electrical stimulation (FES) to achieve substantial outcomes. We therefore recommend a hybrid model: three weekly RAGT sessions (45–60 min) for gait patterning, complemented by two sessions of high-intensity resistance training (60–70% 1RM) on non-training days to address muscle weakness and maximize real-world functional transfer (65).

## 5 Limitations

Several factors inherent to the current body of literature should be considered when interpreting the findings of this meta-analysis. The field of robotic rehabilitation for SCI is still evolving, resulting in a relatively small number of available RCTs, which in turn limits the overall sample size and statistical power for some analyses. The clinical heterogeneity across these studies—regarding patient characteristics, specific robotic devices, and intervention protocols—likely contributed to the moderate statistical heterogeneity observed in the 6MWT outcome. Future multi-center, large-sample studies are warranted to verify whether the observed non-significant improvement in LEMS is a true biological effect or a result of limited statistical power.

Furthermore, while the included studies were generally sound in their randomization, opportunities for methodological enhancement exist. A notable limitation is the current scarcity of long-term follow-up data, as most included studies focused primarily on immediate post-intervention effects. Consequently, it remains unclear whether the observed improvements in 6MWT and WISCI II scores are sustained at 3 or 6 months post-training. Taken together, the GRADE assessment showed that the certainty of evidence ranged from low to moderate, highlighting the need for larger, methodologically rigorous trials with longer follow-up to strengthen the robustness and durability of the current findings.

## Data Availability

The original contributions presented in the study are included in the article/Supplementary material, further inquiries can be directed to the corresponding authors.
