# Supplementary material for: Can robot-assisted gait training improve walking and activity abilities in persons with spinal cord injury? A systematic review and meta-analysis of randomized controlled trials
Source: Front Neurol. 2026 May 29;17:1743421. doi: 10.3389/fneur.2026.1743421 (PMC13262192; doi:10.3389/fneur.2026.1743421)
Supplement: Supplementary file 1 [file Supplementary_file_1.zip › supplementary material/MeSH terms.docx]

**spinal cord injuries**

Injuries, Spinal Cord

Traumatic Myelopathy

Spinal Cord Trauma

Post-Traumatic Myelopathy

Spinal Cord Contusion

Spinal Cord Laceration

Spinal Cord Transection

**Gait**

Gaits

**Robotics**

Remote Operations (Robotics)

Telerobotics

Soft Robotics

Socially Assistive Robots

Social Robots

Humanoid Robots

Companion Robots
